# Targeted Intraoperative Radiotherapy Is Non-inferior to Conventional External Beam Radiotherapy in Chinese Patients With Breast Cancer: A Propensity Score Matching Study

**DOI:** 10.3389/fonc.2020.550327

**Published:** 2020-10-08

**Authors:** Yin Mi, Pengwei Lv, Fang Wang, Lin Li, Mingzhi Zhu, Yanyan Wang, Yingying Zhang, Lele Liu, Qinchen Cao, Meilian Dong, Yonggang Shi, Ruitai Fan, Jingruo Li, Yuanting Gu, Xiaoxiao Zuo

**Affiliations:** ^1^Department of Radiation Oncology, The First Affiliated Hospital of Zhengzhou University, Zhengzhou, China; ^2^Department of Breast Surgery, The First Affiliated Hospital of Zhengzhou University, Zhengzhou, China

**Keywords:** breast cancer, external beam radiotherapy, breast-conserving surgery, intraoperative radiotherapy, Asia

## Abstract

**Purpose:** To investigate the efficacy of targeted intraoperative radiotherapy (TARGIT) vs. conventional external beam radiotherapy (EBRT) in Chinese patients with breast cancer.

**Methods:** We retrospectively analyzed breast cancer patients who underwent breast-conserving surgery (BCS) at our hospital between April 2009 and October 2017. Patients were divided into TARGIT group and EBRT group according to different radiotherapy methods. TARGIT was performed with low-energy X-rays emitted by the Intrabeam system to deliver a single dose of 20 Gy to the applicator surface. Propensity score matching was performed at 1:1. The Kaplan–Meier method was used to calculate the locoregional recurrence (LR), distant metastasis-free survival (DMFS), disease-free survival (DFS), and overall survival (OS) of the two groups, and the log-rank test was run to analyse between-group difference before and after matching.

**Results:** A total of 281 patients were included, with a median follow-up of 43 months. Of them, 82 were included in the TARGIT group and 199 in the EBRT group. Using the risk-adapted approach, 6.1% of patients received supplemental EBRT in the TARGIT group. The 5-year LR rate was 3.2% in the TARGIT group and 3.1% in the EBRT group (*P* = 0.694), the 5-year DMFS rates were 100 and 96.7%, respectively (*P* = 0.157); the 5-year DFS rates were 96.8 and 94.2% (*P* = 0.604); and the 5-year OS rates were 97.6 and 97.8% (*P* = 0.862). After matching which eliminated interference from imbalanced baseline factors, 128 matched patients were analyzed by the Kaplan–Meier method. The 5-year LR rate was 2.3% in the TARGIT group and 1.6% in the EBRT group; the 5-year DMFS rates were 100 and 98.4%, respectively; the 5-year DFS rates were 97.7 and 98.4%; and the 5-year OS rates were 98.4 and 98.4% (*P* = 0.659, 0.313, 0.659, 0.987). There was no significant difference in efficacy between TARGIT group and EBRT group.

**Conclusion:** TARGIT and EBRT have similar 5-year outcomes in selected Chinese breast cancer patients undergoing BCS, and it can be used as an effective alternative to standard therapy, with substantial benefits to patients. The results need to be further confirmed by extending the follow-up time.

## Introduction

In the beginning of the 21st century, long-term follow-up results of prospective studies such as the National Surgical Adjuvant Breast and Bowel Project B-06 study (1–3) showed that for patients with early breast cancer, breast-conserving surgery (BCS) combined with whole-breast external beam radiotherapy (EBRT) is similar to mastectomy with respect to relapse and survival. For about 40 years, BCS plus whole-breast EBRT has been used as the standard treatment for early breast cancer. EBRT usually adopts the conventional segmentation method to deliver a total dose of 45–50 Gy over 5–7 weeks, and most patients require an additional 10–16 Gy to the tumor bed (4). However, in clinical practice, 15 to 30% of patients will decline radiotherapy after BCS (5–8). Some patients even choose to undergo total mastectomy in order to avoid EBRT. Reasons for the low EBRT acceptance include the long EBRT time, high cost, need to travel to treatment centers, and limited mobility (9–11). Some researchers are trying to identify breast cancer patients who do not require postoperative radiotherapy. Based on the inclusion criteria of the Cancer and Leukemia Group B (CALGB) 9,343 and PRIME II studies, few elderly patients with early breast cancer who are eligible for standard endocrine therapy may not require radiotherapy, but they face increased risk of local relapse (12, 13). Many studies have reported that, regardless of whether EBRT was performed, 90% of post-BCS recurrence cases were concentrated in the quadrant of the primary lesions and that the recurrence rate of breast cancer outside the ipsilateral breast tumor bed was similar to that of the contralateral second primary breast cancer (14–16). Whole-breast EBRT may expose the surrounding tissues and organs to radiation, with its associated adverse reactions (17). As a result, some researchers believe that EBRT may be an excessive treatment after BCS.

Targeted intraoperative radiotherapy (TARGIT) employs the Intrabeam system (Zeiss, Germany) to generate low-energy X-rays. During the operation, this method provides all necessary radiation doses under direct vision to target only the tumor bed. Compared with EBRT, this approach allows a much shorter therapy time and a reduced volume of irradiated breast (18).

In 2013 and in 2016, the TARGIT-A trial, a multicentre randomized controlled trial, reported the advantages and disadvantages of TARGIT and EBRT in patients with early breast cancer (19, 20). The TARGIT treatment was non-inferior to the EBRT treatment with respect to overall survival and adverse reactions. However, this conclusion is questioned by some scholars because of the short median follow-up time and high local relapse (21). In 2019, Abo-Madyan et al. (22) reported the results, a single-center study with a median follow-up time of 8.5 years. No significant difference was observed in 5-year local relapse, distant metastasis, or overall survival between the TARGIT group and the EBRT group. While available data are still inadequate to dethrone EBRT as the standard treatment for early breast cancer, TARGIT has shown great potential. Several studies (23–27) have been conducted in Asia to investigate electron intraoperative radiotherapy, but studies on TARGIT are scarce. Previously, we retrospectively analyzed the use of TARGIT in Chinese patients with breast cancer and found that the adverse reactions were tolerable and cosmetic outcomes were good (28). Given this, we further investigated the efficacy of TARGIT vs. EBRT in Chinese patients with breast cancer to explore the value of TARGIT in Asian patients.

## Materials and Methods

### Patient Selection

We retrospectively analyzed the clinical data of all breast cancer patients who underwent BCS at our hospital between April 2009 and October 2017. The decision whether to perform BCS was made by the breast surgeon, radiation therapist, and patient together. Inclusion criteria: maximum tumor diameter <5 cm and patient consent to BCS. The inclusion criteria did not limit lymph node status, hormone receptor status, HER2 status, and tumor grade. The exclusion criteria were as follows: (i) contraindication to radiotherapy or a previous history of radiotherapy in the breast region; (ii) collagen vascular disease; (iii) suspected polycentric lesions based on preoperative mammography, ultrasound, or MRI; (iv) distant metastasis indicated by imaging examination; (v) inflammatory breast cancer; (vi) positive resection margin after extensive local resection of the tumor and failure to ensure a negative margin on subsequent resection; (vii) suspected malignant microcalcification with extensive or diffusive distribution based on imaging; and (viii) pregnancy. A total of 281 breast cancer patients were included in this study. They were divided into two groups: the TARGIT group (a single session of intraoperative radiotherapy in all patients, and additional postoperative EBRT in patients with high risk factors) and the EBRT group (postoperative whole-breast radiotherapy). All patients signed the consent form. This study was approved by the Ethics Committee of the First Affiliated Hospital of Zhengzhou University, China.

### Surgery and Radiotherapy

#### TARGIT Group

BCS in conjunction with TARGIT was performed by professionally trained breast surgeons, radiation therapists, and physical therapists [see Vaidya et al. (29) for details]. Rapid intraoperative pathological examination was performed to ensure that the resection margin was ≥2 mm from the tumor in all directions. An appropriate applicator was selected based on tumor size. A 2.5–3.5 cm spherical applicator was the most commonly used applicator. Intraoperative radiotherapy was performed with low-energy X-rays emitted by the Intrabeam system (Carl Zeiss Surgical, Oberkochen, Germany) to deliver a single dose of 20 Gy to the applicator surface over 15 to 25 min.

#### EBRT Group

As with the TARGIT group, the EBRT group underwent BCS, but not intraoperative radiotherapy. Patients not undergoing chemotherapy were recommended to start EBRT within 4–8 weeks after BCS, and patients undergoing chemotherapy were recommended to start EBRT within 2–4 weeks after the end of chemotherapy. During EBRT, patients were in the supine position, with hands raised above the shoulders. A body mold was used to secure the patient. Computed tomography (CT) was used for positioning and delineation of the target region and organs at risk. If axillary lymph nodes were negative, only the whole breast was irradiated. If positive, the whole breast and affected axillary and supraclavicular/subclavian regions were irradiated. If axillary lymph nodes were positive and the tumor was located in the inner quadrant, the internal mammary lymph node was irradiated while referring to the dose received by the heart and lungs, as appropriate. The tumor bed was delineated based on the lead markers at the surgical scar, and the boost dose was delivered to 1 cm beyond the tumor bed. Radiotherapy was performed with the Axesse linear accelerator (Elekta AB, Stockholm, Sweden) and 6MV-X. The dose was delivered in sequential mode (whole-breast: 46–50 Gy/23–25 fractions; boost dose for tumor bed: 10–14 Gy/5–7 fractions) or concurrent mode (whole-breast: 50.4 Gy/28 fractions; tumor bed: 60.2 Gy/28 fractions). EBRT was performed with intensity-modulated radiation therapy. Cone-beam CT was performed 3 times a week during radiotherapy to reduce radiotherapy errors.

### Postoperative Treatment

Based on clinical data and postoperative pathological data, patients in the TARGIT group received supplementary EBRT (50 Gy/25 fractions; same procedures as the EBRT group; TARGIT replaced external radiation as a tumor bed boost) if the patient had one or more of the following risk factors: age <40, extensive ductal carcinoma *in situ*, invasive lobular carcinoma, positive lymph nodes, extensive lymph vascular space invasion (LVSI), tumor diameter >3 cm, and negative estrogen receptor (ER). We recommended chemotherapy for patients with at least one risk factor: ≥T2, hormone receptor (–), HER2 (+) and tumor grade 3. Endocrine therapy could be performed at the same time as or after radiotherapy. Trastuzumab (3-week cycles, for 1 year) was given as the targeted therapy at the same time as chemotherapy or after chemotherapy. The specific regimen for chemotherapy, endocrine therapy, and targeted therapy was determined based on patient conditions and was given according to standard procedures.

### Follow-Up and Outcome Measures

The date of the patient's surgery in our hospital was used as the starting point of follow-up. Follow-up indicators included locoregional recurrence (LR), distant metastasis-free survival (DMFS), disease-free survival (DFS), and overall survival (OS). Locoregional recurrence was defined as the recurrence of tumors in the ipsilateral breast or affected lymphatic drainage area after BCS. All relapses and metastases were diagnosed by experienced physicians based on physical examination, imaging studies, and pathological data.

### Statistical Analysis

The χ^2^ test or Fisher's exact test was performed to compare general information between the TARGIT group and EBRT group. Propensity score matching (PSM) was performed at 1:1 with a caliper value of 0.03. The variables included age, tumor (T) stage, lymph node (N) stage, ER, progesterone receptor (PR), human epidermal growth factor receptor 2 (HER2), Ki67, tumor grade, histological type, LVSI, chemotherapy, endocrine therapy, trastuzumab therapy, and axillary dissection. The Kaplan-Meier method was used for survival analysis, and the log-rank test was run to analyse between-group difference before and after matching. For plotting the Kaplan–Meier survival curves, data from all patients was used. The log-rank test was also run for univariate analysis of pre-matching covariates. *P* < 0.05 was considered statistically significant. SPSS v22.0 (SPSS Inc., Chicago, IL, USA) was used for statistical analysis.

## Results

### General Characteristics of Patients

A total of 281 female patients with breast cancer who underwent BCS at our hospital between April 2009 and October 2017 met the entry criteria and were included in this study. Of them, 82 were included in the TARGIT group and 199 in the EBRT group. Five (6.1%) patients in the TARGIT group received supplementary EBRT after surgery, and 77 (93.9%) received TARGIT alone. Nine patients (11%) in the TARGIT group underwent lumpectomy at another hospital and were referred to our hospital for second operation and TARGIT based on pathological data. There were no recurrence or death in the nine patients. The incision margin was ≥2 mm from the tumor in all cases. Table 1 shows that significant between-group differences were observed in age, N stage, chemotherapy, and lymph node dissection (all *P* < 0.05). A higher proportion of patients in the EBRT group were <50 years old, had positive lymph nodes, received chemotherapy, and underwent axillary dissection (Table 1). To balance these differences, PSM was performed at 1:1, with 64 patients in each group and no significant between-group difference in general characteristics between the two groups (all *P* > 0.05, Table 2).

**Table 1:** General characteristics of patients in TARGIT group and EBRT group before matching.

**Characteristic**	**TARGIT (*N* = 82), *n* (%)**	**EBRT (*N* = 199), *n* (%)**	***P***
Age (years)			<0.001
<50	21 (25.6)	137 (68.8)	
50–59	21 (25.6)	36 (18.1)	
≥60	40 (48.8)	26 (13.1)	
T stage			0.126
T1	62 (75.6)	132 (66.3)	
T2	20 (24.4)	67 (33.7)	
N stage			0.006
N0	74 (90.2)	146 (73.4)	
N1	7 (8.5)	46 (23.1)	
N2	0 (0)	4 (2.0)	
N3	1 (1.2)	3 (1.5)	
ER			0.356
Positive	66 (80.5)	150 (75.4)	
Negative	16 (19.5)	49 (24.6)	
PR			0.196
Positive	62 (75.6)	135 (67.8)	
Negative	20 (24.4)	64 (32.2)	
HER2			0.484
Positive	12 (14.6)	36 (18.1)	
Negative	70 (85.4)	163 (81.9)	
Ki67 (%)			0.859
<50	61 (74.4)	146 (73.4)	
≥50	21 (25.6)	53 (26.6)	
Tumor grade			0.348
1	6 (7.3)	23 (11.6)	
2	66 (80.5)	144 (72.4)	
3	10 (12.2)	32 (16.1)	
Histology			0.556
IDC	69 (84.1)	171 (85.9)	
DCIS	6 (7.3)	13 (6.5)	
Mixed	5 (6.1)	6 (3.0)	
Other	2 (2.4)	9 (4.5)	
LVSI			1.000
Yes	0 (0)	1 (0.5)	
No	82 (100)	198 (99.5)	
Chemotherapy			0.002
Yes	53 (64.6)	163 (81.9)	
No	29 (35.4)	36 (18.1)	
Endocrine therapy			0.508
Yes	66 (80.5)	153 (76.9)	
No	16 (19.5)	46 (23.1)	
Trastuzumab			0.925
Yes	12 (14.6)	30 (15.1)	
No	70 (85.4)	169 (84.9)	
ALND			<0.001
Yes	9 (11.0)	64 (32.2)	
No	73 (89.0)	135 (67.8)	

*IDC, invasive ductal carcinoma; DCIS, ductal carcinoma in situ; LVSI, lymph vascular space invasion; ALND, axillary lymph node dissection*.

**Table 2:** General characteristics of patients in TARGIT group and EBRT group after matching.

**Characteristic**	**TARGIT (*N* = 64), *n* (%)**	**EBRT (*N* = 64), *n* (%)**	***P***
Age (years)			0.933
<50	21 (32.8)	23 (35.9)	
50–59	20 (31.3)	19 (29.7)	
≥60	23 (35.9)	22 (34.4)	
T stage			0.404
T1	47 (73.4)	51 (79.7)	
T2	17 (26.6)	13 (20.3)	
N stage			0.377
N0	57 (89.1)	55 (85.9)	
N1	6 (9.4)	8 (12.5)	
N2	0 (0)	1 (1.6)	
N3	1 (1.6)	0 (0)	
ER			0.833
Positive	49 (76.6)	50 (78.1)	
Negative	15 (23.4)	14 (21.9)	
PR			0.694
Positive	45 (70.3)	47 (73.4)	
Negative	19 (29.7)	17 (26.6)	
HER2			0.626
Positive	11 (17.2)	9 (14.1)	
Negative	53 (82.8)	55 (85.9)	
Ki67 (%)			0.683
<50	49 (76.6)	47 (73.4)	
≥50	15 (23.4)	17 (26.6)	
Tumor grade			0.277
1	5 (7.8)	9 (14.1)	
2	50 (78.1)	42 (65.6)	
3	9 (14.1)	13 (20.3)	
Histology			0.382
IDC	51 (79.7)	55 (85.9)	
DCIS	6 (9.4)	6 (9.4)	
Mixed	5 (7.8)	1 (1.6)	
Other	2 (3.1)	2 (3.1)	
Chemotherapy			1.000
Yes	42 (65.6)	42 (65.6)	
No	22 (34.4)	22 (34.4)	
Endocrine therapy			0.833
Yes	49 (76.6)	50 (78.1)	
No	15 (23.4)	14 (21.9)	
Trastuzumab			0.626
Yes	11 (17.2)	9 (14.1)	
No	53 (82.8)	55 (85.9)	
ALND			0.611
Yes	8 (12.5)	10 (15.6)	
No	56 (87.5)	54 (84.4)	

*There was no LVSI after matching. IDC, invasive ductal carcinoma; DCIS, ductal carcinoma in situ; ALND, axillary lymph node dissection*.

### Survival Analysis

**Figure 1:**
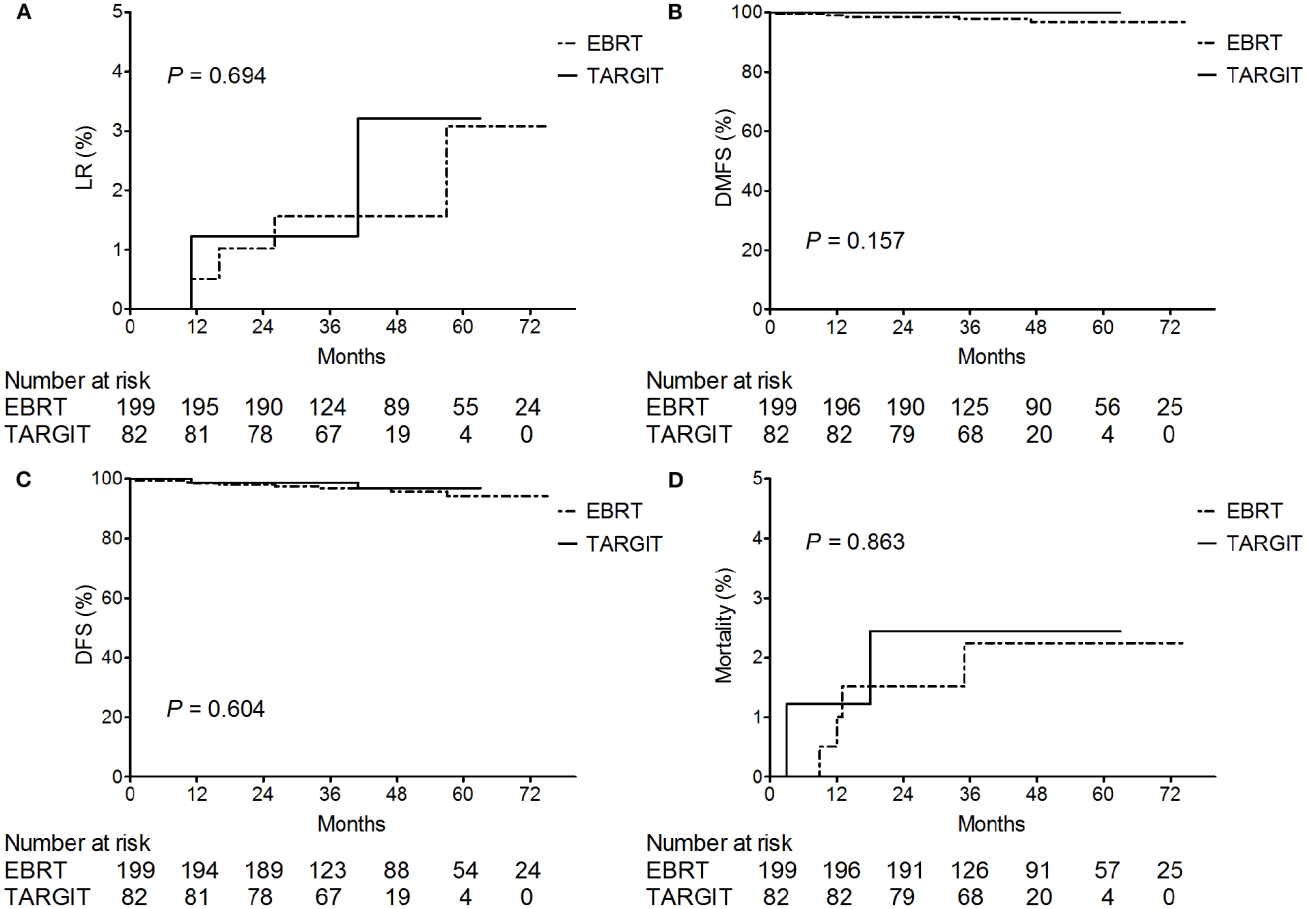
Kaplan–Meier analysis of **(A)** locoregional recurrence, **(B)** distant metastasis-free survival, **(C)** disease-free survival, and **(D)** deaths before matching. For plotting the Kaplan–Meier survival curves, data from all patients was used.

The median follow-up time of 281 patients was 43 months (3–75 months). Before matching, the median follow-up time in the TARGIT group was 44 months, with two cases of local relapse, no distant metastasis, and two deaths; the median follow-up time in the EBRT group was 41 months, with four cases of local relapse, five cases of distant metastasis, and four deaths. Table 3 summarizes the characteristics of patients with locoregional recurrence. Three patients died of breast cancer in the EBRT group, and no patient died of breast cancer in the TARGIT group (Table 4). The 5-year LR rate was 3.2% in the TARGIT group and 3.1% in the EBRT group (*P* = 0.694), the 5-year DMFS rates were 100 and 96.7%, respectively (*P* = 0.157); the 5-year DFS rates were 96.8 and 94.2% (*P* = 0.604); and the 5-year OS rates were 97.6 and 97.8% (*P* = 0.862) (Figure 1). Moreover, no significant between-group difference was observed in breast cancer-related mortality or non-breast cancer-related mortality (*P* = 0.245, 0.154).

**Table 3:** Characteristics of patients with locoregional recurrence.

	**Age (years)**	**T (mm)**	**ER**	**PR**	**HER2**	**Ki67 (%)**	**Histology**	**Grade**	***N***
TARGIT									
	45	27	Negative	Negative	Positive	60	IDC	2	2
	69	30	Positive	Positive	Negative	60	IDC	2	0
EBRT									
	73	17	Negative	Negative	Negative	90	IDC	2	0
	35	20	Negative	Negative	Negative	90	IDC	2	0
	41	22	Negative	Negative	Negative	80	IDC	3	0
	42	25	Positive	Positive	Negative	50	IDC	2	0

*T, Maximum diameter of the tumor; N, Number of metastatic axillary lymph nodes*.

**Table 4:** Causes of death in raw data.

**Causes of death**	**TARGIT (*N* = 82)**	**EBRT (*N* = 199)**	**Total**
Breast cancer	0	3	3
Esophageal cancer	1	0	1
Cardiovascular disease	1	0	1
Pancreatitis	0	1	1
Total	2	4	6

*Data are numbers*.

After PSM (which eliminated interference from imbalanced baseline factors), the median follow-up time was 44 months in the TARGIT group and 34 months in the EBRT group. The Kaplan–Meier method was used to analyse the survival of the 128 matched patients. The 5-year LR rate was 2.3% in the TARGIT group and 1.6% in the EBRT group; the 5-year DMFS rates were 100 and 98.4%, respectively; the 5-year DFS rates were 97.7 and 98.4%; and the 5-year OS rates were 98.4 and 98.4% (*P* = 0.659, 0.313, 0.659, 0.987).

### Univariate Analysis of Pre-matching Data

The log-rank test was performed for univariate analysis of pre-matching covariates (Table 5). The results showed that ER, Ki67, and endocrine therapy were significantly correlated with LR (all *P* < 0.05). T stage and PR was a potential prognostic factor for LR (both *P* < 0.1). N stage was significantly correlated with DMFS (*P* < 0.05), and Ki67 and axillary dissection was a potential prognostic factor for DMFS (both *P* < 0.1). Ki67 was significantly correlated with DFS (*P* < 0.05); T stage, N stage, and axillary dissection was a potential prognostic factor for DFS (all *P* < 0.1). N stage was significantly correlated with OS (*P* < 0.05), and T stage was a potential prognostic factor for OS (*P* < 0.1). We did not perform Cox multivariate analysis because of the small number of outcome-related events.

**Table 5:** Univariate analysis of prognostic factors in 281 breast cancer patients.

**Characteristic**	***n***	**5-year LR (%)**	***P***	**5-year DMFS (%)**	***P***	**5-year DFS (%)**	***P***	**5-year OS (%)**	***P***
**Age (years)**									
<50	158	4.3	0.472	96.7	0.506	92.4	0.268	97.9	0.850
50–59	57	0		100		100		98.2	
≥60	66	3.1		98.5		96.9		97.0	
**T stage**									
T1	194	1.0	0.063	97.7	0.684	97.1	0.053	99.0	0.055
T2	87	8.3		97.3		89.2		95.0	
**N stage**									
N0	220	3.8	0.974	98.5	0.002	95.2	0.072	98.6	0.007
N1	53	4.5		95.5		90.9		95.2	
N2	4	0		100		100		100	
N3	4	0		75.0		75.0		75.0	
**ER**									
Positive	216	2.4	0.016	97.3	0.837	94.9	0.239	97.5	0.700
Negative	65	7.2		98.5		92.8		98.5	
**PR**									
Positive	197	2.7	0.062	97.7	0.649	95.0	0.193	98.0	0.865
Negative	84	5.7		97.3		92.8		97.2	
**HER2**									
Positive	48	3.6	0.943	97.9	0.849	94.3	0.810	97.9	0.984
Negative	233	3.5		97.6		94.5		97.7	
**Ki67 (%)**									
<50	207	0	<0.001	98.9	0.086	98.9	<0.001	98.4	0.182
≥50	74	14.4		94.1		81.4		95.9	
**Tumor grade**									
1	29	0	0.691	100	0.458	100	0.508	100	0.373
2	210	4.0		96.9		93.4		97.0	
3	42	2.6		100		97.4		100	
**Histology**									
IDC	240	4.2	0.779	97.1	0.809	93.4	0.591	97.3	0.791
DCIS	19	0		100		100		100	
Mixed	11	0		100		100		100	
Other	11	0		100		100		100	
**LVSI**									
Yes	1	0	0.903	100	0.901	100	0.870	100	0.880
No	280	3.5		97.6		94.4		97.7	
**Chemotherapy**									
Yes	216	3.4	0.428	98.5	0.318	95.1	0.471	98.5	0.108
No	65	3.4		93.9		92.0		95.4	
**Endocrine therapy**									
Yes	219	2.4	0.012	97.4	0.873	95.0	0.204	97.5	0.739
No	62	7.5		98.4		92.5		98.4	
**Trastuzumab**									
Yes	42	4.0	0.964	97.6	0.764	93.6	0.685	100	0.297
No	239	3.4		97.6		94.6		97.3	
**ALND**									
Yes	73	4.2	0.755	95.4	0.083	91.2	0.093	95.2	0.168
No	208	3.8		98.3		95.1		98.6	
**Mode of radiotherapy**									
TARGIT	82	3.2	0.694	100	0.157	96.8	0.604	97.6	0.862
EBRT	199	3.1		96.7		94.2		97.8	

*IDC, invasive ductal carcinoma; DCIS, ductal carcinoma in situ; LVSI, lymph vascular space invasion; ALND, axillary lymph node dissection*.

## Discussion

For early breast cancer, BCS combined with whole-breast EBRT, along with endocrine therapy, chemotherapy, and targeted therapy as needed, has achieved promising results. Many studies have reported very low local relapse and mortality rates (19, 30–32). In 2009, Botteri et al. (31) analyzed the clinical data of 2,784 patients with early breast cancer treated at the European Institute of Oncology in Milan. All patients underwent BCS and postoperative whole-breast EBRT. The 5-year local relapse rate was 1.1%, and the overall mortality was 3.4%. In 2013, the ELIOT study showed that the 5-year ipsilateral breast tumor recurrence rate was only 0.4% and the mortality was 3.1% after EBRT (32). These studies included patients with high-risk factors, such as positive lymph nodes, negative ER, negative PR, and tumor grade G3. Therefore, the relapse rate and mortality may be lower with more stringent selection.

With the continuous improvement of treatment outcomes, patients are turning their attention to treatment-related adverse reactions, convenience, cost, and cosmetic effects. Some researchers have tried to “subtract” the standard treatment, such as reducing the number of radiotherapy sessions, reducing the area of radiation, and even forgoing radiotherapy in certain breast cancer patients. TARGIT, one of the most popular mobile intraoperative radiotherapy technologies, uses 50-kV low-energy X-rays for direct, single-dose radiation to the tumor bed during operation. Some studies have shown that in general, the side effects of TARGIT are tolerable, the incidence of high-grade side effects is lower than that of conventional EBRT, the local relapse rate and survival rate are non-inferior to those of EBRT, and TARGIT is superior to EBRT in improving the quality of life and cosmetic effects (19, 33–36). However, these studies mainly included non-Asians, with inadequate evidence to support the value of TARGIT in Asian patients with breast cancer. Our previous study showed that TARGIT is safe and feasible in Chinese patients with breast cancer, with few high-grade side effects and good cosmetic effects (28). In this study, we have further confirmed that the efficacy of TARGIT is non-inferior to that of EBRT in selected Chinese patients with breast cancer.

Based on recommendations from the TARGIT-A, ASTRO, and ESTRO studies (19, 37, 38), we selected low-risk patients with breast cancer for TARGIT. Moreover, based on risk-adapted approach from TARGIT-A, patients with risk factors were recommended to undergo EBRT after surgery, and TARGIT was used as a tumor bed boost. The recommended suitability criteria by ASTRO were as follows: age ≥50 years, surgical margin ≥2 mm, Tis or T1, partial ductal carcinoma *in situ*, ER (+), and no LVSI, invasive lobular carcinoma, or other pathological factors (37). As a result, a higher percentage of patients in the EBRT group had risk factors after initial group assignment. In the EBRT group, 68.8% of patients were younger than 50; in the TARGIT group, only 25.6% were. Moreover, 26.6% of patients in the EBRT group had positive lymph nodes; in the TARGIT group, 9.8% did. A higher percentage of patients in the EBRT group received chemotherapy and lymph node dissection. This may be because there were more young patients and lymph node–positive patients in the EBRT group, which affected the treatment choice.

In this study, the overall median follow-up time was 43 months. Kaplan–Meier survival analysis showed no significant between-group difference in LR, DMFS, DFS, or OS. While more patients in the EBRT group had risk factors, chemotherapy, and axillary dissection may help reduce the risks of relapse and metastasis. To balance the differences in baseline factors, PSM was performed at 1:1. Between-group differences in treatment outcomes were still not significant after baseline data matching. These pre-matching and post-matching data demonstrate to certain extent that TARGIT is similar to EBRT in selected Chinese patients with breast cancer.

Some past studies showed that the efficacy of TARGIT was non-inferior to that of EBRT in patients with early breast cancer (19, 22). The TARGIT-A trial (19) enrolled a total of 3,451 patients with breast cancer in 11 countries. The median follow-up time was 2.4 years. The 5-year local relapse rate was 3.3% in the TARGIT group and 1.3% in the EBRT group (*P* = 0.042). The difference did not exceed the pre-defined threshold of 2.5%, so the study concluded that TARGIT was non-inferior to EBRT. The slightly higher relapse rate in the TARGIT group may be related to the enrolment of some high-risk patients who were not ideal candidates for TARGIT. The difference in overall mortality was not statistically significant between the TARGIT group and the EBRT group (3.9 vs. 5.3%, *P* = 0.099). The TARGIT-A trial showed that non-breast cancer–related mortality was significantly lower in the TARGIT group than in the EBRT group (1.4 vs. 3.5%, *P* = 0.0086), which differed from the results of this study. They believe that this is mainly due to the fewer deaths from cardiovascular disease and other tumors in the TARGIT group. Reduced mortality with targeted radiotherapy was also found in two recent meta-analyses (39, 40). However, our study showed no significant between-group difference in non-breast cancer mortality. In the TARGIT group, one patient died of esophageal cancer, and one died of cardiovascular disease. In the EBRT group, only one patient died of pancreatitis. The small sample size may have played a role in these observations. In addition, the patients in the TARGIT group were older (mean age) than the patients in the EBRT group and may have been more susceptible to cardiovascular disease and other tumors. We did not consider the effects of comorbidities when selecting patients, which may have resulted in an imbalance in comorbidities between the two groups. In 2019, a single-center study in Germany extended the median follow-up time to 8.5 years (22). The study included 180 breast cancer patients and found that the 5-year local relapse rate was 0% in the TARGIT group and 1.1% in the EBRT group; the 5-year distant metastasis rates were 3.4 and 2.3%, respectively; and the 5-year OS rates were 94.4 and 93.3% (*P* = 0.317, 0.68, 0.73). The differences were all statistically non-significant. Long-term follow-up data further demonstrated that TARGIT was non-inferior to EBRT in patients with early breast cancer.

BCS without postoperative radiotherapy is unfortunately not uncommon in clinical practice. Tuttle et al. (41) searched the Surveillance Epidemiology and End Results database to analyse breast cancer patients who underwent surgery in the United States between 1992 and 2007 and found that 21.1% of patients did not undergo radiotherapy after BCS and that the percentage of patients choosing not to undergo radiotherapy had risen from 1992 to 2007. Their findings showed that patients at high risk of recurrence were more likely to forgo postoperative radiotherapy. This was also observed in patients who undergo BCS in conjunction with TARGIT. The multi-center retrospective study TARGIT-R in North America showed that some at-risk patients were unwilling to undergo EBRT after TARGIT (33). In this study, 25 at-risk patients in the TARGIT group were recommended to undergo supplementary EBRT, but only five patients did. The main hurdles included the long EBRT time and high cost and that most of these patients may or may not be indicated for intraoperative radiotherapy according to guidelines. Real-world data requires clinicians to follow up these patients closely and provide any necessary remedial treatment in a timely manner. Fortunately, we did not see apparent relapse or metastasis in these patients during the current follow-up period.

We initially planned to incorporate potential prognostic factors (*P* < 0.1) from the univariate analysis into the Cox regression model to identify independent risk factors for treatment outcomes. However, due to overall good treatment results and few outcome-related events, the Cox analysis may have had compromised validity and produced unreliable results. Thus, we did not perform Cox multivariate analysis. The univariate analysis indicated some potential prognostic factors that were reported in previous articles (31, 42, 43). We will continue to extend the follow-up period and observe more outcome-related events to further investigate the effect of each variable on prognosis in Cox analysis.

The small sample size and relatively short follow-up time are main limitations of this study. While the groups were balanced after PSM, some source data were lost in this process. Nevertheless, both pre-matching and post-matching analyses demonstrate that TARGIT is non-inferior to EBRT in selected Chinese patients with breast cancer. The relapse rate, metastasis rate, and mortality are low in Chinese patients undergoing BCS in conjunction with TARGIT. These data suggest that TARGIT is an effective alternative to EBRT in some patients with early breast cancer.

## Conclusion

BCS in conjunction with TARGIT has similar outcomes compared with conventional EBRT in selected Chinese patients with breast cancer. Our results add to international evidence, and support the use of TARGIT in Asian patients with breast cancer, who would benefit from its many advantages such as its great convenience, lower cost, and better quality of life.

## Data Availability Statement

The data analyzed in this study is subject to the following licenses/restrictions: Datasets are available on request. The raw data supporting the conclusions of this article will be made available by the authors, without undue reservation. Requests to access these datasets should be directed to Yin Mi, conankyd@126.com.

## Ethics Statement

The studies involving human participants were reviewed and approved by the Ethics Committee of the First Affiliated Hospital of Zhengzhou University, China. The patients/participants provided their written informed consent to participate in this study.

## Author Contributions

YS, JL, and YG contributed to the conception and design of the study. YW and YZ collected the data. YM analyzed the data and wrote the manuscript. PL, FW, LLi, and MZ did the operation. Intrabeam system used for intraoperative radiotherapy was operated by QC, LLiu, and MD. RF and XZ checked the data and revised the manuscript. All authors read and approved the final manuscript.

## Conflict of Interest

The authors declare that the research was conducted in the absence of any commercial or financial relationships that could be construed as a potential conflict of interest.
